# X-ray micro-computed tomography (*μ*CT): an emerging opportunity in parasite imaging

**DOI:** 10.1017/S0031182017002074

**Published:** 2017-11-28

**Authors:** James D. B. O'Sullivan, Julia Behnsen, Tobias Starborg, Andrew S. MacDonald, Alexander T. Phythian-Adams, Kathryn J. Else, Sheena M. Cruickshank, Philip J. Withers

**Affiliations:** 1 School of Materials, The University of Manchester, Oxford Road, Manchester M13 9PL, UK; 2 School of Biological Sciences, The University of Manchester, Oxford Road, Manchester M13 9PL, UK

**Keywords:** Helminth, microscopy, pathology, X-ray tomography

## Abstract

X-ray micro-computed tomography (*μ*CT) is a technique which can obtain three-dimensional images of a sample, including its internal structure, without the need for destructive sectioning. Here, we review the capability of the technique and examine its potential to provide novel insights into the lifestyles of parasites embedded within host tissue. The current capabilities and limitations of the technology in producing contrast in soft tissues are discussed, as well as the potential solutions for parasitologists looking to apply this technique. We present example images of the mouse whipworm *Trichuris muris* and discuss the application of *μ*CT to provide unique insights into parasite behaviour and pathology, which are inaccessible to other imaging modalities.

## Introduction

Microscopy is a ubiquitous tool for investigating the lifestyles of parasites, and the development of new imaging technologies produces novel insights into how parasites survive in their hosts. For example, electron microscopy captured snapshots of dynamic ultrastructural changes occurring during host invasion by the protozoan *Leishmania donovani* (Loussert *et al.* 2012), whilst the development of fluorescent and luminescent labelled antibodies, as well as transgenic strains of parasites themselves, allow tracking of a spreading infection within a host (Henriques *et al.* 2014; Qin *et al.* 2014; Siciliano and Alano, 2015).

Soil-transmitted helminths infect billions of people worldwide and are a significant economic burden (Pullan *et al.* 2014), yet their lifestyles often remain enigmatic. Complexities surrounding genetic makeup, coupled with difficulties in their maintenance *in vitro*, mean that they lack the potential for genetic tractability which has driven the study of protozoans. An example of these problems is the whipworm *Trichuris* spp., which infects roughly half a billion people worldwide. Existing drugs have limited efficacy (Speich *et al.* 2015), do not prevent reinfection (Jia *et al.* 2012) and concerns have grown that existing drugs may become increasingly redundant in the face of increasing anthelmintic resistance (Wolstenholme *et al.* 2004; Sutherland and Leathwick, 2011; Vercruysse *et al.* 2011). Despite this, little is known about invasion patterns, reproduction and feeding and consequently there is a need for novel approaches to studying the lifestyle of parasites such as *Trichuris* to identify novel therapeutic targets.

Here we present *ex vivo* X-ray micro-computed tomography (*μ*CT) as a largely unexploited opportunity which can avoid the limitations of other prominent imaging modalities and provide new insights into parasite lifestyle. Whilst *μ*CT has existed for over 30 years (Elliott and Dover, 1982), relatively recent progress in optimizing imaging technology and soft tissue sample preparation has vastly improved the accessibility of the technique for researchers interested in studying parasites. As well as detailing the working principles of *μ*CT, we also summarize aspects of sample preparation which we deem of importance to those looking to visualize samples of soft tissue. Example images of the mouse whipworm *Trichuris muris*, which has an unusual ‘intracellular’ lifestyle embedded within the gut lining of its murine host, are used to illustrate the unique ability of *μ*CT to visualize the spatial positioning of parasites embedded within the host tissue *ex vivo*, without any need for sectioning. As well as minimizing sectioning-associated artefacts, we also discuss how analysis of virtual slices in three-dimensional (3D) data presents unique advantages over other imaging modalities, and how 3D images provide novel opportunities for education and engagement, including *via* 3D printing.

## Principles of X-ray *μ*CT

**Fig. 1.**
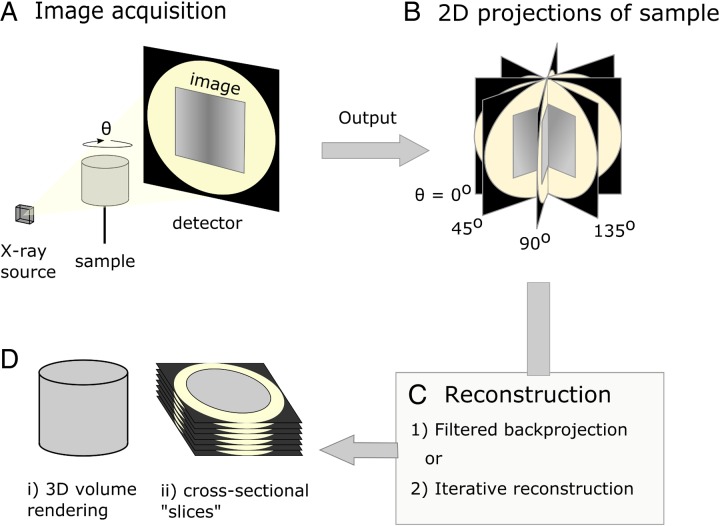
Diagrammatic representation of X-ray micro-computed tomography workflow. (A) Basic illustration of tomographic apparatus, including the X-ray source, detector and sample. Projection images are made as the sample is rotated at increments through *θ*. (B) The raw output of the tomogram is a series of projections of the sample taken at different angles. (C) Projections are digitally ‘reconstructed’; two commonly used approaches are filtered backprojection and iterative reconstruction. Reconstruction algorithms output a dataset which is suitable for analysis. (D) The sample may be viewed in a virtual environment in a variety of ways, including as a 3D rendered volume (Di). Alternatively, 2D cross-sections, or ‘slices’ (Dii), of the sample may be viewed.

X-ray *μ*CT was developed in the early 1980s (Dover *et al.* 1981; Elliott *et al.* 1981; Elliott and Dover, 1982) and could be described as the microscopic cousin of the clinical computed tomography (CT) scanners used to examine human patients in hospitals today. However, whereas medical scanners usually have resolutions in the mm range, *μ*CT systems typically achieve resolutions in the 1–100 *µ*m range. When imaging using X-ray tomography, many (typically between 500 and 3000) 2D projections (digital radiographs) of a sample are collected from many different angles using penetrating X-rays. Following their acquisition, a 3D image of the absorptive power of the sample is reconstructed computationally from the 2D projections (Fig. 1). Tomographic images can also be reconstructed from images produced with gamma rays (Ter-Pogossian *et al.* 1975), neutrons (Winkler, 2006), electrons (Crowther *et al.* 1970) or visible light (Sharpe *et al.* 2002). However, X-rays are often chosen based on the balance between penetration of, and attenuation by, the sample, such that sufficient **contrast** (**glossary**, Box 1) can permit the differentiation of internal features of interest. The contrast results from the differential attenuation of X-rays, as determined by the **attenuation coefficient** of the constituent materials. If a material is dense, or includes heavy elements, more X-rays will be attenuated and **transmittance** will be reduced. Upon acquisition of the data, the 3D volume can be interrogated by examining key virtual cross-sectional images or the whole image stack. In many cases quantification of features in the imaged volume requires the application of ‘segmentation’ workflows, a process by which domains/features of interest within the sample are virtually distinguished and labelled. Subsequently, visualization and quantitative analysis of the number, morphology and distribution of the features can then be undertaken (Maire and Withers, 2014).

Box 1. Glossary

**Attenuation coefficient:** Characterizes how easily a volume of a material may be penetrated by a beam of photons

**Coherence:** A quality of waves on the electromagnetic spectrum, such that highly coherent X-rays have the same phase difference and frequency

**Contrast:** The difference in intensity between an image and its direct background relative to overall background intensity

**Geometric magnification:** Magnification which is produced by varying the ratio of distances between the X-ray source, sample and detector. A smaller distance between the source and sample, and larger distance between the sample and detector, increases the magnifying effect

**Spatial resolution:** The smallest distance between two structures such as they may be distinguished by the observer to be separate

**Tomogram:** A reconstructed tomographic image

**Transmittance:** The ratio of light falling on a body which passes through it

There are at least two scanning arrangements by which the necessary projections are acquired. The first involves rotating a gantry-mounted X-ray source and detector around the object. This method is used in clinical CT scanners and has the advantage for *in vivo* study that the subject is easily stabilized on a flat surface, minimizing soft tissue organ movement. The **spatial resolution** in the gantry-based scanners typically used in preclinical research involving rodents approaches 50 *µ*m (Ritman, 2011). The second scanning arrangement involves rotating the sample on a table whilst keeping the source and detector stationary (see Fig. 1A). Instruments based on the rotating table approach often exploit the use of X-ray lenses and/or variable **geometric magnification** to generate images typically with superior spatial resolutions, which can reach sub-micron levels in certain instruments (Withers, 2007). Rotating table *μ*CT has found usage in a broad range of research areas, including materials (Stock, 2008), geology (Cnudde and Boone, 2013) and life science (Bradley and Withers, 2016).

*In vivo* preclinical studies have typically used gantry-based scanning arrangements to image whole model organisms such as mice (Cunha *et al.* 2014). Provided that sufficient contrast can be achieved, *in vivo* studies of parasites offer exciting avenues for future study, particularly for time-lapse monitoring of infection (Lee *et al.* 2007; Dillon *et al.* 2013) and assessment of pathology (Ha *et al.* 2016). Whilst rotating table systems have been used for preclinical *in vivo* studies on rodent models (Paulus *et al.* 2000; Burstein *et al.* 2002), they are now commonly used to study inanimate objects and *ex vivo* systems. *Ex vivo* tissue samples are amenable to a range of sample preparation techniques including staining which provide the necessary contrast for viewing histological features. Furthermore, longer image acquisitions facilitating higher spatial resolutions are possible *ex vivo*, as X-ray dose does not need to be limited as with living organisms (Ford *et al.* 2003). Within the last decade, the ability to retrieve 3D images at close to histological resolution means rotating table *μ*CT has attracted an increasing amount of attention from a broader range of biologists, notably palaeontologists (Tafforeau *et al.* 2006) and comparative morphologists (Metscher, 2009*a*), but largely not yet parasitologists.

In this paper, we will consider the opportunities recently opened up by high-resolution *ex vivo μ*CT with regard to its potential application to parasitological questions. Since its inception, there have been a multitude of methodological developments in *μ*CT technology for biological imaging, exhaustive discussion of which is beyond the scope of this article. However, correlative tomography (Burnett *et al.* 2014), in which results of multiple imaging modalities are combined, may be of interest to those attempting to integrate functional information in a larger spatial context. In ‘correlative workflows’, *μ*CT datasets have been combined with light microscopic histological observations (Duke *et al.* 2009; Particelli *et al.* 2012; Bagi *et al.* 2015; Geffre *et al.* 2015) and electron micrographs (Handschuh *et al.* 2013; Bushong *et al.* 2015). Such multiscale studies put functional and structural observations on a histological or subcellular scale into the larger spatial context provided by *μ*CT.

## Contrast enhancement for parasite imaging

### Use of contrast agents

**Fig. 2.**
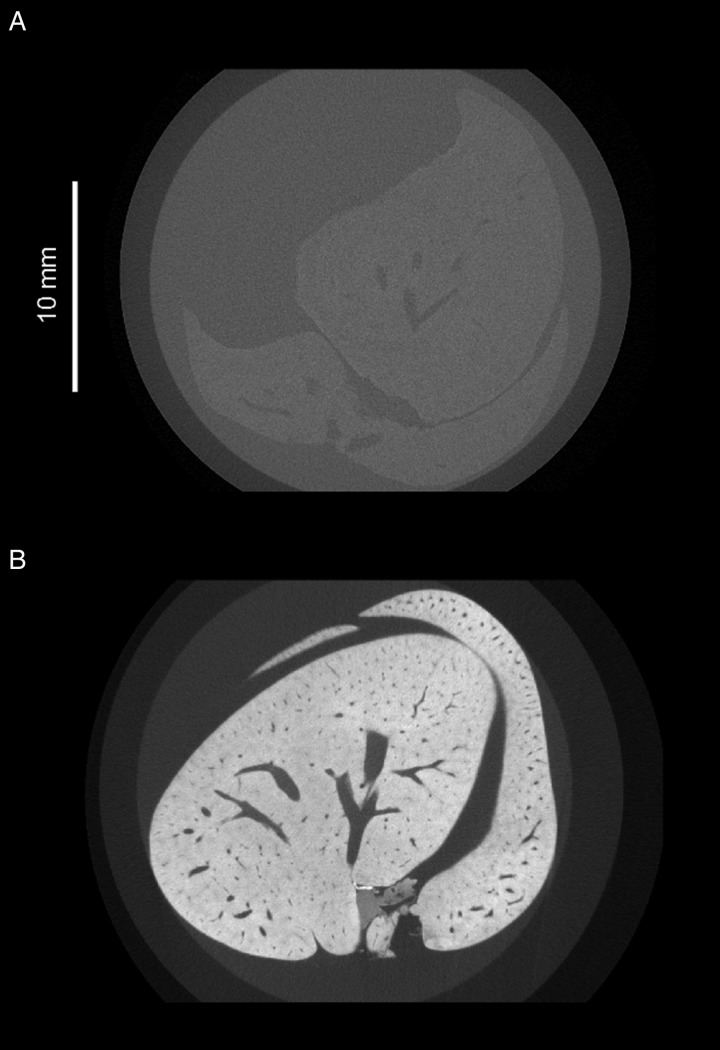
Greyscale slices of two whole mouse livers, acquired under the same X-ray beam energies but differentially prepared. (A) Unstained mouse liver. The outline of the liver is barely visible, and little internal detail can be distinguished. (B) Mouse liver which has been immersed in a mixture containing 1·66% m/v I2 and 3·44% KI for 48 h. Liver tissue appears brighter due to increased attenuation of X-rays from the source, and there is high contrast between the vascular lumen and the surrounding tissue.

Imaging parasites within soft tissue introduces challenges for the tomographic process. Because soft tissue is low-density and consists mostly of light elements such as carbon and hydrogen, it attenuates X-rays poorly, resulting in low contrast between internal features. Staining with a suitable contrast agent can greatly enhance visibility of tissue structure (Fig. 2). In the case of *μ*CT, ideal stains incorporate heavy elements which bind differentially to internal regions of a sample and provide increased contrast due to higher range of X-ray attenuation. Iodine (as aqueous I_2_KI or in ethanol) is a popular choice of stain among researchers looking to visualize soft tissue organization (Gignac *et al.* 2016). Stains traditionally used for electron microscopy such as osmium tetroxide and uranyl acetate have also been adopted, as their high electron densities have a translatable attenuating effect on transmitted X-rays (Descamps *et al.* 2014), and their common usage between *μ*CT and electron microscopy facilitates correlative workflows. Staining agents must be able to penetrate the whole way into a sample, and therefore staining agents are generally chosen based on fast diffusion rates, rather than specific binding properties. Systematic assessment of a variety of staining species has identified potassium iodide (KI) and mercuric chloride as demonstrating suitably fast diffusion rates (Pauwels *et al.* 2013). However, phosphotungstic acid and phosphomolybdenic acid, which have a slower rate of diffusion, have been highlighted as effective in visualization of collagenous structures due to their specific binding properties (Metscher, 2009*b*; Nierenberger *et al.* 2015).

It is important to note that the optimal staining time and concentration will be unknown in soft tissues which have not been previously investigated, as differences in tissue composition will result in varying stain-binding affinities and rates of stain uptake. A recent meta-analysis of iodine staining includes comprehensive sample preparation information for a large variety of soft tissue samples of different sizes obtained from different species, which authors may refer to in order to ease the amount of guesswork required for previously unstudied samples (Gignac *et al.* 2016). However, no similar meta-analyses are available for other stains, which may require a trial-and-error approach using multiple scans to determine the optimal stain concentration and staining time (Aslanidi *et al.* 2013). An additional consideration is the risk of sample shrinkage during the immersion staining process, with the degree of shrinkage increasing with the concentration of the staining solution (Vickerton *et al.* 2013). However, treatment in graded ethanol before staining is efficient in minimizing shrinkage and preserving the native structure of tissue (de Souza e Silva *et al.* 2015).

### Phase contrast

Phase contrast is an alternative contrast-enhancement method originally developed for light microscopy, the physical principles of which have since been exploited in *μ*CT instruments. Whilst unstained biological soft tissue is weakly absorbing of the hard energy X-rays produced by laboratory sources, it can produce significant shifts in the phase of transmitted X-rays. Phase contrast is the term used to describe imaging which exploits this effect. For most laboratory *μ*CT systems, the illuminating X-rays show little **coherence** and phase gratings must be used to generate phase contrast in the image (Momose *et al.* 2003). However, some laboratory systems do exhibit sufficient coherence for propagation-based imaging (Wu, 2014), edge illumination (Olivo and Speller, 2007) or Zernike phase contrast (Bradley *et al.* 2010). Consequently, phase contrast techniques are now more accessible to researchers trying to image soft tissue without the use of staining (Bech *et al.* 2009; Zamir *et al.* 2016). Indeed, grating and propagation-based phase contrast have both been investigated as alternatives to serial light microscopic histology (Zanette *et al.* 2013; Holme *et al.* 2014).

## What parasitological questions could *μ*CT address?

Until now, a great deal of morphological information about helminths has been gathered by both scanning and transmission electron microscopy, which boast sub-micron resolutions superior to those achievable by *μ*CT systems (Egerton, 2005*a*, *b*). However, a sample prepared for *μ*CT does not need to be physically sectioned to view internal structure, so information about the spatial positioning of parasites relative to the host can be gathered without the risk of sectioning-associated artefacts. Furthermore, unusual features are more likely to be found because a volume is interrogated, rather than a cross-sectional area such as a tissue section, which may or may not intersect with the feature of interest. Volumes can be subsequently examined at a higher resolution by electron microscopy within a correlative tomography framework if required. In the case of *Trichuris*, imaging a 3D volume also uniquely allows trajectories of multiple worms to be separated in relation to surrounding host tissue. However, similarly to techniques involving sectioning, rotating-table *μ*CT at histological (i.e. 1 *µ*m) resolution is generally applicable only to *ex vivo* fixed samples for several reasons. Firstly, the time required to acquire all the projections for *μ*CT, typically between 2 and 10 hours using a laboratory source, precludes imaging of dynamic processes on a similar timescale [although a small number of studies have imaged slow (occurring over several days (Lowe *et al.* 2013)) or cyclical processes (Mokso *et al.* 2015) *in vivo*, albeit in comparatively radiation-tolerant insects]. Additionally, the short distance between the X-ray source and sample (in the orders of a few millimetres to 10s of millimetres) required to achieve the highest resolutions in a region of interest is only practical for *ex vivo* samples. The low contrast provided by unstained tissue is also a factor which precludes imaging of soft tissue *in vivo* at histological resolution. Magnetic resonance imaging (MRI), which is amenable to *in vivo* imaging, has been used to analyse parasite-induced pathology in whole animals (Voieta *et al.* 2010; Masi *et al.* 2015). However, MRI has a spatial resolution approaching 25–100 *µ*m in the most powerful magnetic fields (Shapiro *et al.* 2004; de Kemp *et al.* 2010), and no meaningful morphological information about tissue structure can be gathered below this threshold.

The first ever published micro-computed **tomogram** depicted the snail *Biomphalaira glabrata*, a vector of the blood fluke *Schistosoma mansoni* (Elliott and Dover, 1982). However, to date, *μ*CT has been applied minimally in parasitological contexts. It has been used to investigate the internal head morphology of *Rhodinus prolixus*, the insect vector for Chagas disease, the imaging of which is facilitated by the relatively high X-ray attenuation of the insect's chitinous cuticle (Sena *et al.* 2014). The unique capabilities of *μ*CT represent an unexploited opportunity to visualize the spatial positioning of helminth endoparasites and their associated pathology. The technique also possesses key advantages over light and electron microscopy in the context of visualizing an organism embedded within soft tissue. A key potential application of *μ*CT is that 3D imaging facilitates assessment of accumulation and distribution of parasites within whole organs. This capability has been exploited by two helminth-centric studies to our knowledge, including visualization of cyst accumulation of the trematode *Paragonimus westermani* in the lung of dogs and an accompanying description of connective channels between the cysts and the surrounding bronchi (Lee *et al.* 2007). Heterogeneous distribution of Schistosome eggs in the vertebrate central nervous system has also been described (Bulantová *et al.* 2016). Such examples highlight the potential of 3D data to explore phenomena such as migration and invasion of host tissue through the tracking of multiple parasites in relation to the host tissue at once. Higher resolution *μ*CT has also served to highlight interactions between protozoan parasites and host tissues on the nanoscale. In *Plasmodium falciparum*, 3D visualization of parasite-infected erythrocytes has allowed quantification of their changing volume and haemoglobin content throughout the course of infection (Hanssen *et al.* 2012), as well as collapse of membrane integrity before egress of merozoites (Hale *et al.* 2017).

**Fig. 3.**
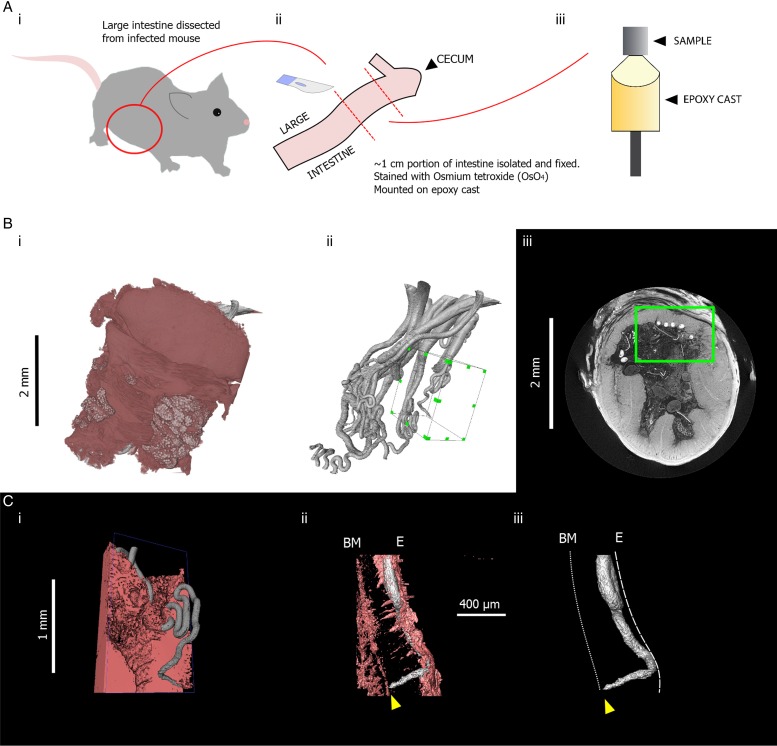
Summary of sample preparation protocol and representative X-ray tomography images of *Trichuris muris*, followed by images acquired on an Xradia Versa XRM 520 tomograph, highlighting positioning of a *Trichuris* head. (Ai) The large intestine was dissected from a *T. muris*-infected mouse. (Aii) An approximately 1 cm length of the dissected gut was isolated, fixed in 4% PFA, stained with osmium tetroxide (OsO_4_) and (Aiii) mounted on an epoxy cast. (Bi) 3D volume rendering of the gut section containing *Trichuris* and (Bii) 3D surface rendering of the worms that were embedded in that gut section. A green-highlighted cuboid indicates a region of interest which includes the head of a single worm. (Biii) a green square shows the position of the same region of interest in a 2D slice. (Ci) a pseudocoloured volume-rendering of a subsection of the region of interest including *Trichuris* (grey) embedded within the gut lining (pink). (Cii) Virtual 3D ‘surfaces’ showing the positioning of the head of *Trichuris* (grey) in relation to the gut lining (pink). The tip of the head is marked by a yellow arrow. (Ciii) The gut lining is virtually removed from the image, showing the positioning of the tip of the head in relation to the planes of the basement membrane (BM) and the internal epithelial surface (E), which are indicated by dotted white lines.

Apart from requiring a large time investment when investigating a large volume, several other disadvantages also accompany the use of manual sectioning. Samples must often be cut down to a size appropriate for the sectioning equipment, and given a specific orientation so that features of interest identified may be visualized optimally. Such procedures result in a risk of scientifically novel features, which are unaccounted for in the study design, being overlooked. To illustrate how *μ*CT can reduce this risk, we include an example where the *μ*CT workflow captures positioning of the anterior-most portions of *Trichuris* towards the basement membrane of the gut lining (Fig. 3), which is an observation made much more accessible by the capability of *μ*CT to visualize the trajectory of an entire worm. Furthermore, finding parasites within a pre-cut block of tissue is a difficult undertaking with no guarantee of success, as researchers are blind to the internal structure pre-sectioning. In *μ*CT, virtual slices of any orientation within an unsectioned sample can be visualized, and regions of interest can be determined in a more informed manner once the internal structure is known.

**Fig. 4.**
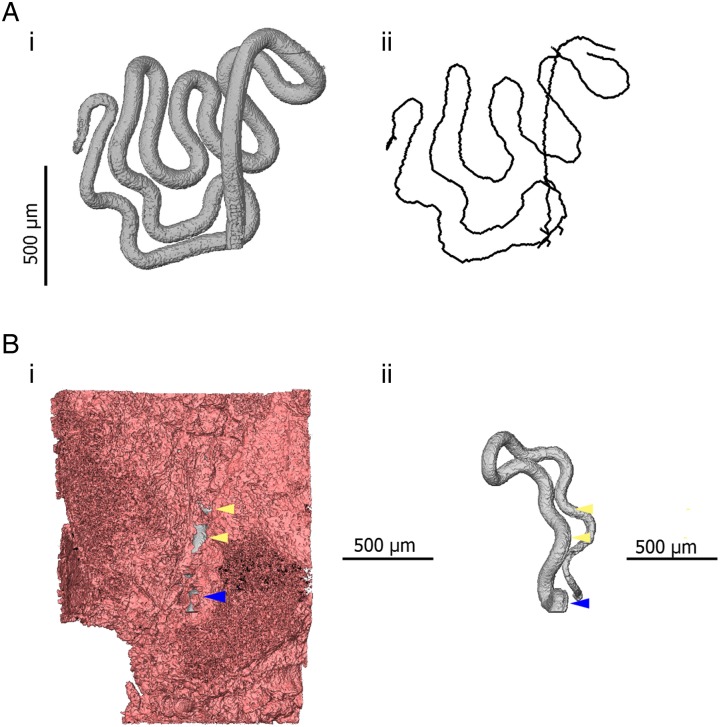
3D models indicating quantitative measurements possible in 3D datasets. (Ai) 3D model of the anterior of *Trichuris* (grey) which has been segmented (virtually distinguished and labelled, so that it can be visualized independently of surrounding tissue) using the AVIZO visualization software. (Aii) A spatial graph is shown, which is produced from an algorithm designed to find and measure the centreline of filamentous structures. In this way, the length of a portion of a worm, for instance, which embedded within the gut lining, can be measured. (Bi) 3D models of the anterior of *Trichuris* (grey) embedded in the epithelium (pink); (Bii) is the same image, with the epithelium virtually removed, such that only the embedded worm is visible. Blue arrows indicate the position at which the worm ‘enters’ the gut lining, and yellow arrows indicate breaks or tears in epithelium overlying the embedded worm. The proportion of the embedded worm which is exposed by these breaks can be estimated by using the ‘centreline tree’ algorithm.

The use of *μ*CT also introduces new options for quantitative measurement. Similarly to digitally stored histology slides, 2D distance measurements can be made in virtual slices of a sample. Additionally, existing software and algorithms designed for the analysis of non-biological *μ*CT data can be co-opted for the study of parasites. One example of this specific to *Trichuris* is the use of skeletonization algorithms which estimate the length and topology of filamentous structures. The visualization and analysis software AVIZO (FEI Visualization Sciences Group) includes propriety skeletonization algorithms. However, the Fiji distribution of ImageJ (Schindelin *et al.* 2012) also includes a skeletonization extension based on the algorithm of Lee *et al.* (1994). Skeletons produced in this way can then be analysed using the Analyze Skeleton extension developed for ImageJ (Arganda-Carreras *et al.* 2010). In the case of *Trichuris*, skeletonization can be employed to estimate the length of a worm which is embedded within the gut lining (Fig. 4). Furthermore, the length of a given parasite which is covered by the epithelium and the overall integrity of the epithelial niche can be estimated. Such inferences may have importance in determining required pharmacokinetic properties of drugs; if integrity of the epithelial tunnel is high, drugs can be designed to have local epithelial penetrance; in contrast, if large proportions of the worm are exposed to the gut lumen by ‘breaks’ in the epithelial tunnel such novel drug design may not be necessary.

## Concluding remarks

Whilst X-ray *μ*CT has been only minimally used by parasitologists to date, its increasing availability and performance presents a variety of new research opportunities. As a non-destructive method, when coupled with measures taken to enhance image contrast during sample preparation and imaging, the ability to track multiple parasites within intact host tissue particularly provides new opportunities. This technique has already been exploited by a small number of studies for investigating parasite accumulation, distribution and migration, as well as pathology. Qualitative and quantitative analysis of data collected from such efforts is already enabled by a broad array of existing software tools. Additionally, publically archived 3D datasets provide an excellent opportunity to continue data analysis post-publication and can constitute an exciting medium for education and public engagement through the medium of 3D printing.
